# Living with mother alone: exploring the risk of stunting among under-five children in India

**DOI:** 10.3389/fnut.2025.1700655

**Published:** 2025-12-02

**Authors:** Soumen Barik, Anuj Singh, Mayank Singh

**Affiliations:** 1 Department of Fertility & Social Demography, International Institute for Population Sciences (IIPS), Mumbai, India; 2 Department of Sciences (Mathematics), Indian Institute of Information Technology (IIIT), Ranchi, Jharkhand, India; 3 Department of Epidemiology and Biostatistics, KAHER, Belgaum, Karnataka, India

**Keywords:** single-mother households, child stunting, structural inequity, maternal height, wealth index, children under 5, India

## Abstract

**Background:**

Stunting affects nearly one in three children under five in India. While socioeconomic, maternal, and environmental factors are well-established determinants, the role of household structure particularly children living with only their mother remains underexplored. Therefore, the present study aims to examine the association between family structure and stunting among children under 5 years of age in India. Specifically, the objective is to compare the risk of stunting among children living with their mothers only to those living with both parents and to identify the key factors contributing to any observed disparities.

**Data and methods:**

This study analyzed data from 224,218 surviving children aged 0–59 months from the NFHS-5 (2019–21), which is a cross-sectional survey conducted across India and is nationally representative for all key indicators of women and child health. The main outcome of interest stunting was defined as height-for-age Z-score < −2 SD. Children were classified by living arrangement: with both parents or mother alone. Multivariable logistic regression models were sequentially adjusted for child, maternal, household, and community-level factors. Furthermore, Fairlie decomposition analysis quantified the contribution of observed characteristics to stunting disparities.

**Results:**

A total of 1.35% of children lived with their mother alone, among whom stunting prevalence was significantly higher (39.8%) compared to those with both parents (35.43%). In unadjusted models, child with mother alone was associated with 21% higher odds of stunting (OR: 1.21; 95% CI: 1.12–1.30). However, this association gradually weakened after accounting for confounders in the fully adjusted model (OR: 1.07; 95% CI: 0.97–1.18). Fairlie decomposition (model III) revealed that 70.19% of the stunting gap was attributable to observed factors. Household wealth was the largest contributor (28.29%), followed by maternal height (24.23%) and social caste (13.01%), indicating that structural inequities, rather than the family structure per se, drive the observed disparity.

**Conclusion:**

The elevated stunting risk among children living with mothers alone is not intrinsic to single-mother households but is largely associated by socioeconomic disadvantage, maternal undernutrition, and systemic inequities. Policies targeting poverty reduction, maternal health, and education are essential to address stunting in vulnerable family settings.

## Introduction

Stunting, defined as impaired linear growth for age, is characterized by a height-for-age measurement that falls at least two standard deviations below the median of the WHO Child Growth Standards (1). This condition primarily results from inadequate dietary intake of energy and essential nutrients necessary to support optimal growth and development (2). Stunting serves as a key indicator of chronic undernutrition, affecting millions of children globally and posing a significant threat to their health and long-term well-being. Compared to their non-stunted peers, stunted children face an elevated risk of mortality and are more susceptible to life-threatening infectious diseases, including diarrhea, pneumonia, malaria, and measles (3). The condition is particularly prevalent among children exposed to recurrent infections, those residing in socioeconomically disadvantaged households, and those born to mothers who consumed inadequate diets before and during pregnancy (4). The long-term consequences of stunting extend beyond physical growth, encompassing impaired cognitive development, reduced educational achievement, and consequently diminished economic productivity in adulthood (5). Moreover, stunting is associated with an increased risk of overweight and non-communicable diseases later in life, thereby contributing to the dual burden of malnutrition (5).

Addressing malnutrition in all its forms is intrinsically linked to international human rights instruments that affirm the universal right to food. Article 25 of the Universal Declaration of Human Rights explicitly recognizes the right to an adequate standard of living sufficient to ensure health and well-being, including access to adequate and nutritious food (6). In the recants estimate in 2024, 150.2 million children under the age of five (23.2%) were affected by stunting (7). In the context of India, there has been a significant drop in stunting in the last two decades (8). Stunting reduced from 51.9% in NFHS-1 to 34.1% in NFHS-5 still stunting is persistent within the population (9). Previous studies have highlighted multiple determinants of stunting, including poverty, maternal education, inadequate infant and young child feeding practices, poor sanitation, and recurrent infections (10, 11). However, the role of household structure, particularly the absence of one parent, has received far less attention in India.

In India, evolving family structures—shaped by demographic transition, modernization, and urbanization have introduced new complexities to the challenge of child undernutrition (12). Rising divorce rates (13), the rapid shift from traditional joint families to nuclear households driven by increasing literacy, economic transformation, and greater autonomy for women (12), and the growing prevalence of single-parent households (14, 15), collectively reflect profound changes in familial and social organization. These transformations mirror broader shifts in marriage and partnership dynamics across the country (16). Empirical evidence underscores this trend: Dommaraju (17) documents a marked decline in joint family living arrangements alongside a rise in one-person and nuclear households, fueled by factors such as higher educational attainment, employment in the formal sector, declining fertility, and rural-to-urban migration. More recently, Chakravorty et al. (18) emphasized that these evolving family structures are deeply intertwined with wider socioeconomic, cultural, and behavioral transformations in Indian society.

Children living with only their mother due to widowhood, divorce, or separation are at heightened risk of undernutrition. Single mothers, especially those facing economic hardship, often struggle to provide adequate nutrition due to limited financial resources, restricted healthcare access, and economic instability. Although children in single-mother households are more likely to be undernourished than those in two-parent families (19). Research has largely focused on broad determinants like household income and maternal education (20–22). While a few African studies have differentiated between types of single motherhood—such as widowhood, divorce, or non-marital childbearing in relation to child nutrition (23). This nuance is largely absent in the Indian context (24, 25). Consequently, the specific pathways through which diverse forms of single motherhood affect child nutritional outcomes in India remain underexplored (26). This knowledge gap limits the development of effective interventions to address the economic and social challenges faced by single-mother households. Financial hardship, social stigma, and the emotional burdens of single motherhood can adversely affect children’s nutritional status and overall health (27). Importantly, different forms of single motherhood may exert distinct influences on child nutrition: unmarried mothers often lack spousal financial support, heightening economic insecurity, while divorced or widowed mothers may experience greater psychological stress—both pathways potentially increasing the risk of child stunting (14). Addressing these disparities aligns with the United Nations Sustainable Development Goals (SDGs), particularly Goal 2 (zero hunger, food security, and improved nutrition) and Goal 3 (healthy lives and well-being for all). This study investigates the association between single motherhood and child stunting in India and decomposes the disparity in stunting outcomes between children living with their mother alone and those living with both parents, aiming to elucidate the unique challenges faced by single-mother households. This study aims to inform evidence-based policies and targeted interventions that improve child health outcomes and reduce the burden of undernutrition among this vulnerable population.

## Data and methods

This study utilizes data from the fifth round of the National Family Health Survey (NFHS-5), conducted in 2019–21 across all states and union territories in India. NFHS-5 is a nationally representative, cross-sectional survey that collects extensive information on population health, fertility, child nutrition, mortality, family planning and maternal and child healthcare utilization. The analysis is restricted to children aged 0–59 months for whom complete anthropometric, household, and maternal information was available.

**Figure 1:**
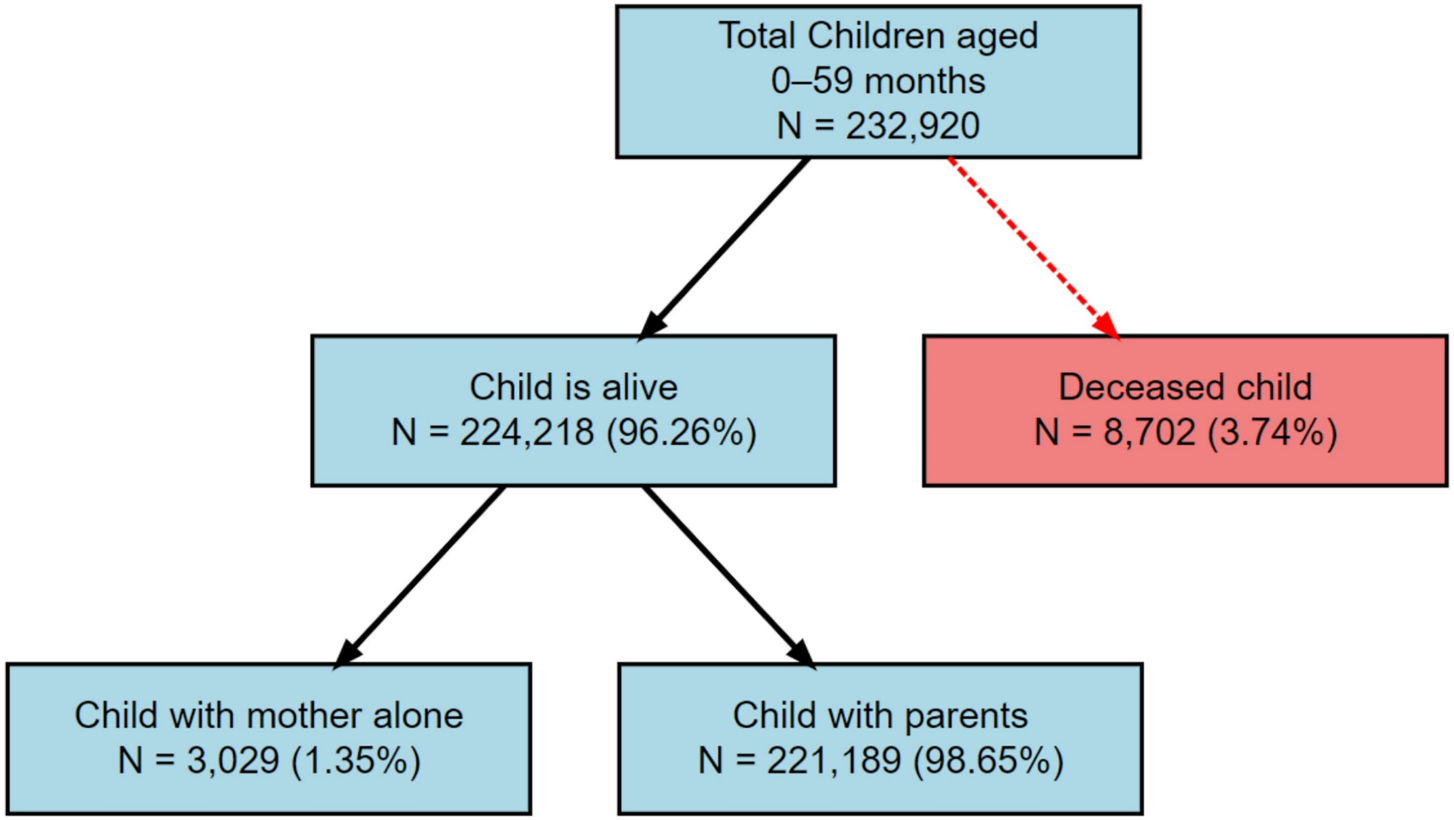
Diagrammatic representation of the stepwise sample selection procedure.

Figure 1 illustrates the sample selection process of the analytical sample from the total pool of children aged 0–59 months available in the NFHS dataset (*N* = 232,920). The first stage excluded children who were not alive at the time of survey. A total of 8,702 children (3.74%) were deceased and therefore not included in further analysis. The remaining 224,218 children (96.26%) were eligible for analysis. Among the surviving children, classification was done based on household composition. Children were categorized as either living with both parents or with their mother alone, based on co-residence information. The majority of children (*N* = 221,189; 98.65%) were living with both parents. Only a small fraction (*N* = 3,029; 1.35%) were living with their mother alone.

### Variable description

The primary outcome variable is stunting, defined as a child having a height-for-age Z-score (HAZ) less than −2 standard deviations from the WHO reference population. The HAZ scores were pre-calculated in the NFHS dataset using WHO child growth standards. A binary variable was created to distinguish between stunted (HAZ < −2) and non-stunted children (HAZ ≥ −2).

The main explanatory variable is child’s living arrangement, categorized as:

1. Child living with both parents (reference group), and
2. Child living with mother only.

To account for potential confounding factors, a wide range of covariates were included, grouped into child, maternal, household, and community-level characteristics: Child-level variables include sex of the child, age in months (categorized in 15-month intervals), birth order, size at birth (mother’s perception), and birth weight (classified as low or normal). Maternal characteristics consist of age at childbirth, education level, body mass index (BMI), height, age at first birth, number of antenatal care (ANC) visits, place of delivery (institutional or non-institutional), and pregnancy wantedness. Household and community factors encompasses the wealth index (based on household assets), household size, caste group (SC/ST, OBC, others), religion (Hindu, Muslim, Christian, others), place of residence (urban/rural), region (North, South, East, West, Central, Northeast), sex of the household head, distance to healthcare facility, exposure to mass media, and family planning messages.

### Statistical analysis

Descriptive statistics were computed to present the background characteristics of the sample by living arrangement and their association with stunting status. Pearson’s chi-square test was used to assess the significance of associations.

To assess the association between living arrangement and risk of stunting, a series of logistic regression models were estimated. Model 1 includes unadjusted odds ratios (UOR). Model 2 adjusts for child-level characteristics. Model 3 includes child and maternal characteristics. Model 4 is the full model adjusted for child, maternal, household, and community characteristics.

The binary logistic is usually put into a more compact form as follows:


$$\text{logit}(Y)=\ln(\frac{P}{1-P})=\alpha +\beta_{1}x_{1}+\beta_{2}x_{2}+\beta_{3}x_{3}\ldots \ldots \ldots \ldots .+\beta_{k}x_{k}+\varepsilon .$$

Where, $\beta_{1},\beta_{2,}\ldots \ldots \ldots ,\beta_{k}$ are regression coefficients indicating the relative effect of a particular explanatory variable on outcome. The coefficients change as per the context in the analysis.

In order to quantify the contribution of observed differences in characteristics to the gap in stunting between children living with both parents and those living with their mother only, a Fairlie decomposition technique was applied. This non-linear decomposition method is appropriate for binary outcomes and extends the Blinder-Oaxaca decomposition to logistic regression frameworks. The method estimates the proportion of the observed difference in stunting that can be explained by group differences in the distribution of explanatory variables. Three decomposition models were applied:

1. Model with child characteristics only
2. Model including child and maternal characteristics
3. Full model including child, maternal, household, and community-level characteristics.

Mathematically, the Fairlie decomposition for two groups A and B can be expressed as:


$$\bar{Y_{A}}–\bar{Y_{B}}=[\frac{1}{N_{A}}\;\sum_{i=1}^{N_{A}}F(X_{\mathrm{Ai}}\hat{\beta })-\frac{1}{N_{B}}\;\sum_{i=1}^{N_{B}}F(X_{\mathrm{Bi}}\hat{\beta })\;]$$

Where $\bar{Y_{j}}$ denotes the average predicted probability of stunting in group j, X is a vector of covariates, $\hat{\beta }\;$are coefficient estimates from the pooled logit model, and F (·) is the cumulative logistic function. All the analysis has been done with STATA 16.0 version and also used weight for all the analysis.

## Results

Table 1 shows the distribution of background characteristics and the prevalence of stunting among under-five children by living arrangement and other covariates.

**Table 1:** Background characteristics and stunting prevalence among under-five children by household structure, India (NFHS-5, 2019–21).

Background characteristics	Sample (%)	Nutritional status		*p* value
		Non-stunting	Stunting	
Child characteristics				
Child status				
Child with parents	229,754 (98.95)	131,306 (64.57)	71,989 (35.43)	<0.001
Child with mother	3,166 (1.05)	1,637 (60.2)	1,083 (39.8)	
Sex of the child				
Male	120,665 (51.96)	67,454 (63.75)	38,857 (36.25)	<0.001
Female	112,255 (48.04)	65,489 (65.36)	34,215 (34.64)	
Age of the child				
0–15 months	62,119 (26.67)	39,159 (72.97)	14,523 (27.03)	<0.001
16–30 months	57,171 (24.6)	31,078 (60.24)	20,270 (39.76)	
31–45 months	57,291 (24.59)	30,963 (60.53)	20,366 (39.47)	
46–59 months	56,339 (24.14)	31,743 (63.92)	17,913 (36.08)	
Birth order				
First	89,139 (39.12)	53,523 (68.56)	24,850 (31.44)	<0.001
Second/Third	113,034 (48.91)	64,409 (63.76)	36,285 (36.24)	
Fourth and above	30,747 (11.97)	15,011 (54.47)	11,937 (45.53)	
Size of child at birth				
Small	23,492 (10.75)	11,864 (58.48)	8,294 (41.52)	<0.001
Average	163,929 (70.31)	95,185 (65.21)	50,938 (34.79)	
Large	41,954 (18.94)	24,430 (65.79)	12,645 (34.21)	
Birth weight				
Low birth weight	77,881 (38.73)	40,632 (59.64)	27,428 (40.36)	<0.001
Optimal birth weight	131,378 (61.27)	81,305 (69.03)	37,335 (30.97)	
Maternal/self-characteristics				
Women age				
15–24	71,946 (32.95)	40,163 (64.09)	22,832 (35.91)	<0.001
25–34	138,035 (58.77)	79,828 (64.95)	42,856 (35.05)	
More then 35	22,939 (8.28)	12,952 (63.2)	7,384 (36.8)	
Educational status				
No education	51,210 (21.36)	24,554 (53.65)	19,937 (46.35)	<0.001
Primary	30,081 (12.32)	15,506 (58.39)	11,024 (41.61)	
Secondary	119,864 (50.69)	71,305 (66.66)	35,480 (33.34)	
Higher	31,765 (15.64)	21,578 (77.08)	6,631 (22.92)	
ANC visit				
<=3	73,048 (40.75)	41,457 (63)	23,598 (37)	<0.001
>3	101,435 (59.25)	62,493 (68.59)	29,151 (31.41)	
Delivery place				
Non-institutional	31,609 (11.2)	15,537 (53.82)	12,130 (46.18)	<0.001
Institutional	201,311 (88.8)	117,406 (65.85)	60,942 (34.15)	
BMI of women				
Normal	132,544 (61.01)	78,172 (65.22)	41,804 (34.78)	<0.001
Underweight	40,539 (19.93)	20,776 (56.91)	15,754 (43.09)	
Overweight/Obese	37,418 (19.06)	24,997 (73.12)	9,104 (26.88)	
Mather height				
Short (<145)	26,408 (12.1)	10,923 (46.32)	12,453 (53.68)	<0.001
Medium (145–154.9)	135,635 (59.75)	77,193 (63)	45,417 (37)	
Normal/Tall (≥155)	64,736 (28.15)	44,096 (75.45)	14,764 (24.55)	
Age at mother first birth				
Less_than_20	76,017 (34.15)	40,054 (31.79)	26,882 (38.33)	<0.001
20_to_29	148,748 (62.89)	87,659 (64.94)	44,235 (59.38)	
30_to_49	8,155 (2.95)	5,230 (3.26)	1955 (2.29)	
Pregnancy wantedness				
Wanted	213,046 (90.83)	122,027 (64.85)	66,424 (35.15)	<0.001
Unwanted	19,874 (9.17)	10,916 (61.3)	6,648 (38.7)	
Community and household characteristics				
Exposure to mass media				
No	67,768 (28.27)	33,721 (24.29)	25,556 (34.76)	<0.001
Any	165,152 (71.73)	99,222 (75.71)	47,516 (65.24)	
Distance to health facility				
No problem	85,125 (39.74)	51,065 (41.91)	24,275 (35.92)	<0.001
Problem	147,795 (60.26)	81,878 (58.09)	48,797 (64.08)	
Exposure to family planning massage				
No	64,533 (26.86)	33,831 (58.96)	22,537 (41.04)	<0.001
Yes	168,387 (73.14)	99,112 (66.53)	50,535 (33.47)	
Household members				
1_to_4	60,455 (25.57)	34,775 (66.06)	17,888 (33.94)	<0.001
5_to_8	135,558 (57.64)	77,492 (64.13)	43,407 (35.87)	
More_than_8	36,907 (16.78)	20,676 (63.59)	11,777 (36.41)	
Sex of the household				
Male	197,535 (84.82)	113,265 (64.79)	61,419 (35.21)	<0.001
Female	35,382 (15.18)	19,675 (63.05)	11,653 (36.95)	
Wealth index				
Poorest	63,406 (24.59)	30,629 (53.88)	25,010 (46.12)	<0.001
Poorer	54,463 (21.73)	29,629 (60.33)	18,643 (39.67)	
Middle	45,083 (19.54)	26,613 (65.59)	13,566 (34.41)	
Richer	39,094 (18.41)	25,048 (71.8)	9,713 (28.2)	
Richest	30,874 (15.73)	21,024 (77.1)	6,140 (22.9)	
Social caste				
SC/ST	94,966 (34.86)	51,663 (60.38)	32,679 (39.62)	<0.001
OBC	89,093 (45.52)	50,772 (64.98)	27,585 (35.02)	
Other	38,178 (19.61)	24,083 (70.41)	9,586 (29.59)	
Religion				
Hindu	171,055 (79.41)	97,097 (64.54)	53,903 (35.46)	<0.001
Muslim	33,522 (16.24)	18,765 (63.18)	10,444 (36.82)	
Christian	18,851 (2.07)	11,155 (68.37)	6,177 (31.63)	
Other	9,492 (2.28)	5,926 (69.71)	2,548 (30.29)	
Residence				
Urban	47,199 (26.65)	29,100 (69.84)	12,255 (30.16)	<0.001
Rural	185,721 (73.35)	103,843 (62.64)	60,817 (37.36)	
Region				
East	45,227 (26.13)	24,278 (61.34)	15,860 (38.66)	<0.001
West	20,552 (12.44)	11,491 (63.37)	6,830 (36.63)	
North	43,090 (13.4)	27,065 (70.45)	11,178 (29.55)	
South	29,269 (16.52)	17,655 (70.34)	8,012 (29.66)	
Central	60,560 (27.88)	32,044 (61.68)	20,208 (38.32)	
Northeast	34,222 (3.63)	20,410 (64.84)	10,984 (35.16)	

### Child characteristics

In the analytic sample (*N* = 232,920; children living with both parents = 229,754, 98.95%; children living with mother only = 3,166, 1.05%), the overall prevalence of stunting was higher among children living with their mother only (39.8%) than among those living with both parents (35.43%; χ^2^, *p* < 0.001).

By sex, 36.25% of males and 34.64% of females were stunted (*p* < 0.001). Stunting varied with child age: prevalence was lowest in the youngest group (0–15 months, 27.03%) and highest for children aged 16–45 months (≈39.5% for 16–30 and 31–45 months). Children of higher birth order (≥4) showed higher stunting (45.53%) compared with firstborns (31.44%; *p* < 0.001). Children perceived as small at birth had higher stunting (41.52%) than those perceived average or large (p < 0.001), and low birth weight children had greater stunting (40.36%) than children with optimal birth weight (30.97%; *p* < 0.001).

### Maternal / self-characteristics

Stunting prevalence varied substantially across maternal characteristics. By maternal age, children of mothers aged 15–24 and 25–34 had similar stunting prevalence (~35–36%), while those of mothers ≥35 years showed 36.8% stunting (*p* < 0.001). Maternal education showed a clear gradient: children of mothers with no education experienced the highest stunting (46.35%), compared with 33.34% for secondary and 22.92% for higher education (*p* < 0.001). Children of underweight mothers (BMI < 18.5) had higher stunting (43.09%) than those of normal (34.78%) or overweight mothers (26.88%; *p* < 0.001). Short maternal stature was strongly associated with child stunting: mothers <145 cm had 53.68% of children stunted versus 24.55% among mothers ≥155 cm (*p* < 0.001).

### Community and household characteristics

Community and household context were also strongly associated with stunting. Children in poorest households had stunting of 46.12% compared with 22.9% in the richest quintile (*p* < 0.001). Children in rural areas had higher stunting (37.36%) than urban children (30.16%; *p* < 0.001). Exposure to mass media, distance to health facility, exposure to family planning massage, household size, caste and region showed significant bivariate associations with stunting (all *p* < 0.001). Regional differences were notable: Central and Eastern regions had higher stunting (~38–39%), while North and South showed lower prevalence (~29–30%; *p* < 0.001).

**Figure 2:**
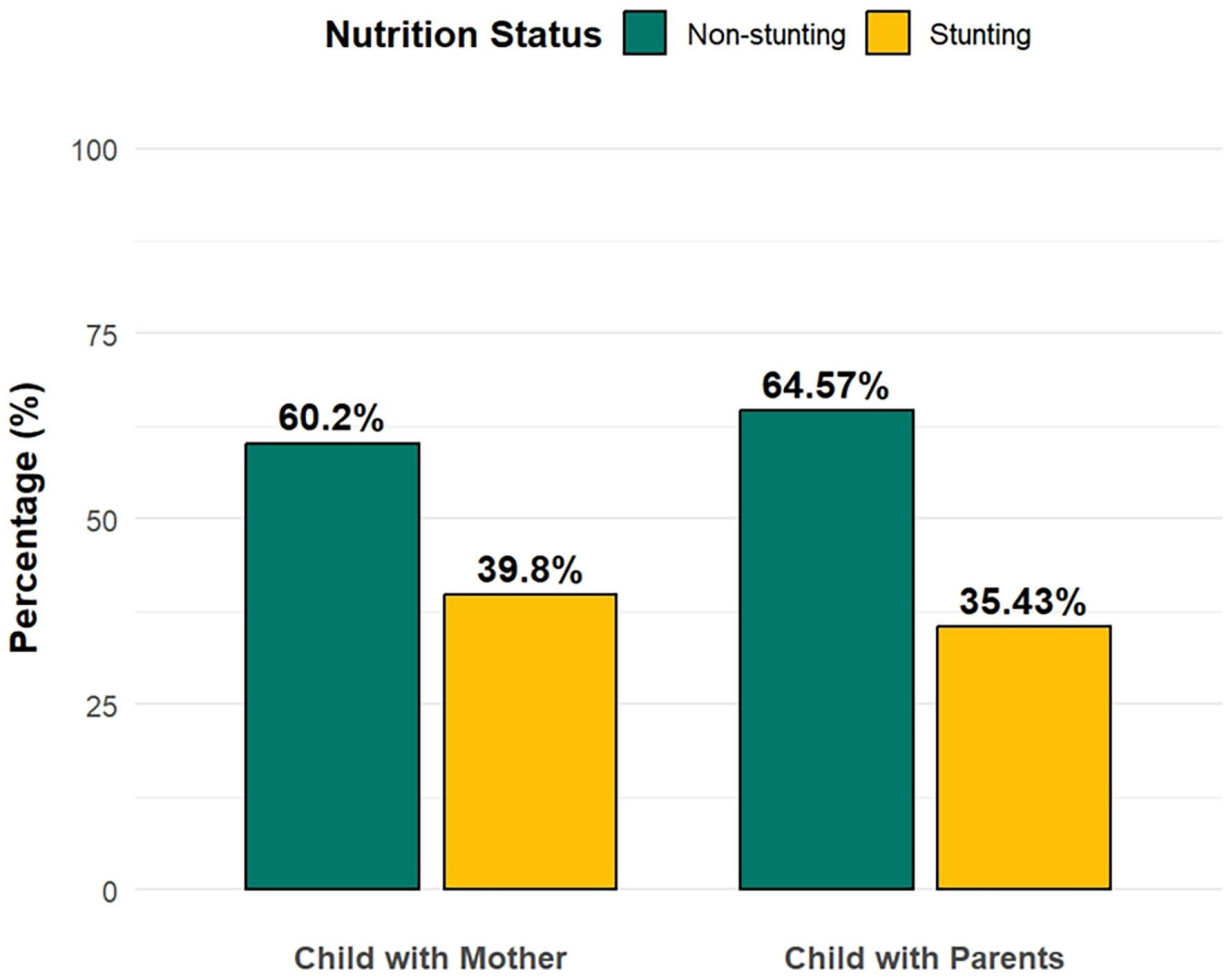
Prevalence of stunting among children living with mother only versus those living with both parents. Comparison of stunting and non-stunting percentages between children living with both parents and those living with mother only. Weighted percentages based on NFHS data.

Figure 2 illustrates the prevalence of stunting among under-five children by living arrangement. The figure clearly shows that children living with their mother only experience a higher prevalence of stunting (39.8%) compared with those living with both parents (35.43%). Conversely, the proportion of non-stunted children is lower among those living with mother only (60.2%) compared to their counterparts living with both parents (64.57%). This pattern indicates a measurable nutritional disadvantage among children residing with their mother alone.

Table 2 presents the logistic regression estimates of the association between child living arrangement and stunting among under-five children in India across four nested models. In the unadjusted model (Model 1), children living with their mothers only had 21% higher odds of being stunted compared to those living with both parents (UOR = 1.21; 95% CI: 1.12–1.30; *p* < 0.001). After adjusting for child-level characteristics, including age, sex, and birth order (Model 2), the association remained statistically significant but slightly attenuated (AOR = 1.17; 95% CI: 1.07–1.27; *p* < 0.001). With the inclusion of maternal characteristics such as education, body mass index (BMI), height, antenatal care visits, and place of delivery (Model 3), the association was further reduced but continued to be statistically significant (AOR = 1.11; 95% CI: 1.01–1.22; *p* < 0.05). However, in the fully adjusted model (Model 4), which incorporated household and community-level variables—such as wealth index, caste, religion, residence, region, household size, exposure to mass media, and accessibility to health facilities—the association between living with mother alone and stunting became statistically non-significant (AOR = 1.07; 95% CI: 0.97–1.18; *p* > 0.05). This finding indicates that the initial association between living arrangement and stunting is largely explained by socioeconomic and contextual factors rather than family structure itself.

**Table 2:** Logistic regression estimates of the association between living with mother alone and stunting among under-five children in India.

Background characteristics	Model 1 (UOR)	Model 2 (AOR)	Model 3 (AOR)	Model 4 (AOR)
Child characteristics				
Child status				
Child with parents				
Child with mother	1.21*** (1.12,1.30)	1.17*** (1.07,1.27)	1.11** (1.01,1.22)	1.07 (0.97,1.18)
Sex of the child				
Male				
Female		0.89*** (0.87,0.90)	0.85*** (0.83,0.87)	0.85*** (0.83,0.87)
Age of the child				
0–15 Months				
16–30 Months		1.74*** (1.69,1.79)	1.77*** (1.71,1.82)	1.81*** (1.75,1.87)
31–45 Months		1.72*** (1.67,1.76)	1.71*** (1.65,1.77)	1.77*** (1.71,1.83)
46–59 Months		1.47*** (1.43,1.51)	1.43*** (1.38,1.49)	1.48*** (1.42,1.54)
Birth order				
First				
Second/Third		1.21*** (1.19,1.24)	1.19*** (1.16,1.23)	1.15*** (1.12,1.19)
Fourth and above		1.70*** (1.65,1.76)	1.47*** (1.40,1.54)	1.36*** (1.29,1.44)
Size of child at birth				
Small				
Average		0.85*** (0.82,0.88)	0.87*** (0.84,0.91)	0.86*** (0.83,0.90)
Large		0.84*** (0.81,0.87)	0.86*** (0.82,0.90)	0.83*** (0.79,0.87)
Birth weight				
Low birth weight				
Optimal birth weight		0.69*** (0.67,0.70)	0.75*** (0.73,0.77)	0.76*** (0.74,0.78)
Maternal/self-characteristics				
Women age				
15–24				
25–34			0.88*** (0.85,0.91)	0.90*** (0.87,0.93)
More then 35			0.79*** (0.75,0.84)	0.82*** (0.77,0.87)
Educational status				
No education				
Primary			0.94*** (0.90,0.98)	0.98 (0.94,1.02)
Secondary			0.77*** (0.74,0.80)	0.86*** (0.83,0.90)
Higher			0.62*** (0.59,0.65)	0.77*** (0.73,0.81)
ANC visit				
<=3				
>3			0.98 (0.96,1.01)	0.98 (0.96,1.01)
Delivery place				
Non-Institutional				
Institutional			0.91*** (0.87,0.95)	0.93*** (0.88,0.97)
BMI of women				
Normal				
Underweight			1.34*** (1.30,1.38)	1.28*** (1.24,1.32)
Overweight/Obese			0.77*** (0.74,0.79)	0.81*** (0.78,0.84)
Mather height				
Short (<145)				
Medium (145–154.9)			0.56*** (0.54,0.59)	0.57*** (0.55,0.59)
Normal/Tall (≥155)			0.35*** (0.34,0.36)	0.36*** (0.35,0.38)
Age at mother first birth				
Less_than_20				
20_to_29			0.98 (0.95,1.01)	0.99 (0.96,1.02)
30_to_49			0.98 (0.91,1.06)	1 (0.92,1.09)
Community and household characteristics				
Pregnancy wantedness				
Wanted				
Unwanted			0.97 (0.93,1.02)	0.99 (0.95,1.04)
Exposure to mass media				
No				
Any				0.95*** (0.92,0.98)
Distance to health facility				
No problem				
Problem				1.01 (0.98,1.04)
Exposure to family planning massage				
No				
Yes				1.00 (0.97,1.03)
Household members				
1_to_4				
5_to_8				1.03** (1.00,1.06)
More_than_8				1.07*** (1.03,1.12)
Sex of the household				
Male				
Female				1.02 (0.98,1.05)
Wealth index				
Poorest				
Poorer				0.92*** (0.89,0.95)
Middle				0.82*** (0.78,0.85)
Richer				0.71*** (0.67,0.74)
Richest				0.64*** (0.60,0.67)
Social caste				
SC/ST				
OBC				0.93** (0.90, 0.95)
Other				0.82*** (0.79,0.86)
Religion				
Hindu				
Muslim				1.15*** (1.10,1.20)
Christian				1.01 (0.95,1.07)
Other				0.93** (0.87,1.00)
Residence				
Urban				
Rural				0.99 (0.95,1.02)
Region				
East				
West				1.36*** (1.30,1.43)
North				1.12*** (1.07,1.17)
South				1.24*** (1.18,1.30)
Central				1.13*** (1.09,1.18)
Northeast				0.97 (0.92,1.03)

*Odds ratios (OR) with 95% confidence intervals; significance levels: *p < 0.05; **p < 0.01; and ***p < 0.001.

In the fully adjusted model, several covariates were found to be significantly associated with stunting. Female children were less likely to be stunted compared to male children (AOR = 0.85; 95% CI: 0.83–0.87). The risk of stunting increased consistently with child’s age, with children aged 16–30 months (AOR = 1.81; 95% CI: 1.75–1.87), 31–45 months (AOR = 1.77; 95% CI: 1.71–1.83), and 46–59 months (AOR = 1.48; 95% CI: 1.42–1.54) showing significantly higher odds of being stunted compared to those aged 0–15 months. Higher birth order was also associated with increased risk—second or third order (AOR = 1.15; 95% CI: 1.12–1.19) and fourth or higher order (AOR = 1.36; 95% CI: 1.29–1.44). Children with an average (AOR = 0.86; 95% CI: 0.83–0.90) or large size at birth (AOR = 0.83; 95% CI: 0.79–0.87) and those with optimal birth weight (AOR = 0.76; 95% CI: 0.74–0.78) were significantly less likely to be stunted than children of small size or low birth weight.

Regarding maternal characteristics, the results demonstrated strong protective effects of maternal education. Children of mothers with secondary (AOR = 0.86; 95% CI: 0.83–0.90) and higher education (AOR = 0.77; 95% CI: 0.73–0.81) were considerably less likely to be stunted compared with those whose mothers had no education. Maternal nutritional status also emerged as a key predictor: children of underweight mothers were more likely to be stunted (AOR = 1.28; 95% CI: 1.24–1.32), while those of overweight or obese mothers had reduced odds (AOR = 0.81; 95% CI: 0.78–0.84). Maternal height was inversely associated with stunting; children of mothers with medium height (145–154.9 cm) had lower odds (AOR = 0.57; 95% CI: 0.55–0.59), and those of tall mothers (≥ 155 cm) had the lowest odds (AOR = 0.36; 95% CI: 0.35–0.38). Furthermore, institutional delivery was significantly protective against stunting (AOR = 0.93; 95% CI: 0.88–0.97).

At the household and community level, the odds of stunting were slightly higher among children from larger households, with 5–8 members (AOR = 1.03; 95% CI: 1.00–1.06) and more than 8 members (AOR = 1.07; 95% CI: 1.03–1.12) compared to those from smaller households. A distinct socioeconomic gradient was observed across the wealth index, with decreasing odds of stunting among children from richer households: poorer (AOR = 0.92; 95% CI: 0.89–0.95), middle (AOR = 0.82; 95% CI: 0.78–0.85), richer (AOR = 0.71; 95% CI: 0.67–0.74), and richest (AOR = 0.64; 95% CI: 0.60–0.67) compared with the poorest households. Caste-based differences were also evident, as children from “Other” caste groups were less likely to be stunted than those belonging to Scheduled Castes and Scheduled Tribes (AOR = 0.82; 95% CI: 0.79–0.86). By religion, Muslim children had slightly higher odds of stunting than Hindu children (AOR = 1.15; 95% CI: 1.10–1.20). Regional disparities persisted even after full adjustment, with children from the Central (AOR = 1.13; 95% CI: 1.09–1.18), Western (AOR = 1.36; 95% CI: 1.30–1.43), Northern (AOR = 1.12; 95% CI: 1.07–1.17), and Southern (AOR = 1.24; 95% CI: 1.18–1.30) regions exhibiting higher odds of stunting compared to those from the Eastern region.

Table 3 presents the Fairlie decomposition results explaining the gap in stunting prevalence between children living with both parents and those living with their mothers only. The decomposition sequentially includes child, maternal, and household-level characteristics to identify the major factors contributing to the observed difference in stunting between the two groups.

**Table 3:** Fairlie decomposition of the gap in stunting prevalence between children living with both parents and those living with mother only in India.

Background characteristics	Child characteristics contribution (Model I)			Child and women characteristics contribution (Model II)			Child, women and household characteristics contribution (Model III)		
	Coefficient	SE	Percent contribution	Coefficient	SE	Percent contribution	Coefficient	SE	Percent contribution
Child characteristics									
Sex of the child	−0.0002	0.00003	1.65	0.0001	0.00003	0.53	0.0002	0.00003	−0.55
Age of the child	−0.0094	0.00032	99.89	−0.0151	0.00060	−55.73	−0.0165	0.00064	47.86
Birth order	−0.0007	0.00014	7.53	0.0020	0.00021	7.38	0.0014	0.00022	−4.16
Size of child at birth	−0.0003	0.00007	3.33	−0.0002	0.00007	−0.83	−0.0002	0.00008	0.63
Birth weight	0.0013	0.00005	−14.06	−0.0005	0.00004	−1.71	−0.0006	0.00005	1.76
Maternal/self-characteristics									
Women age				0.00192	0.00028	7.06	0.0019	0.00032	−5.39
Educational status				−0.00605	0.00029	−22.27	−0.0030	0.00030	8.73
ANC visit				0.00000	0.00003	0.00	0.0000	0.00003	−0.03
Delivery place				−0.00117	0.00031	−4.30	−0.0009	0.00032	2.75
BMI of women				−0.00107	0.00010	−3.94	−0.0007	0.00011	2.00
Mather height				−0.00695	0.00017	−25.56	−0.0084	0.00020	24.23
Age at mother first birth				−0.00013	0.00018	−0.48	−0.0001	0.00019	0.29
Pregnancy wontedness				0.00008	0.00007	0.31	0.0000	0.00009	−0.04
Community and household characteristics									
Exposure to mass media							−0.0003	0.00010	0.79
Distance to health facility							−0.0001	0.00008	0.18
Exposure to family planning massage							0.0000	0.00014	0.03
Household members							0.0007	0.00024	−2.07
Sex of the household							−0.0012	0.00145	3.40
Wealth index							−0.0098	0.00058	28.29
Social caste							−0.0045	0.00056	13.01
Religion							0.0017	0.00128	−4.91
Residence							0.0001	0.00009	−0.15
Region							0.0057	0.00109	−16.39
Explained (%)	21.09			55.12			70.19		
Unexplained (%)	78.91			44.88			29.81		

In Model I, which included only child characteristics, approximately 21.09% of the observed difference in stunting was explained by the included variables, while 78.91% remained unexplained. Among child-level factors, the age of the child emerged as the dominant contributor, accounting for nearly all of the explained portion (coefficient = −0.0094; ≈99.9% contribution). This suggests that differences in age composition between the two groups substantially drive the observed stunting gap. Other child-level variables such as sex, birth order, size at birth, and birth weight made smaller notable contributions.

In Model II, which incorporated both child and maternal characteristics, the proportion of the stunting gap explained increased markedly to 55.12%, with the unexplained component declining to 44.88%. The inclusion of maternal variables significantly enhanced the model’s explanatory power. Among these, maternal height and maternal education emerged as the most influential contributors. Maternal height had a large negative coefficient (−0.00695), explaining approximately 25.6% of the gap, while maternal education contributed 22.3%. These findings indicate that disparities in maternal nutritional status and educational attainment are key mediators of the difference in stunting between children living with both parents and those living with their mothers only. Maternal BMI and place of delivery also contributed modestly to explaining the gap, although their effects were smaller in magnitude.

In Model III, which additionally accounted for household and community-level characteristics, the explained share further increased to 70.19%, leaving 29.81% of the gap unexplained. The inclusion of socioeconomic and contextual variables substantially improved the explanatory capacity of the model. Among these, household wealth index (coefficient = −0.0098; contribution = 28.3%) and social caste (coefficient = −0.0045; contribution = 13.0%) were the strongest contributors, reflecting the central role of socioeconomic inequality in driving nutritional disparities. Other contextual factors, such as region (−16.4%) and religion (−4.9%), also contributed to the explained portion, while exposure to mass media and household size showed relatively minor effects.

## Discussion

This study examined the association between living arrangements—specifically, whether a child lives with both parents or only with the mother—and the risk of stunting among children under five in India, using nationally representative data from the fifth round of the National Family Health Survey (NFHS-5, 2019–21). The analysis included 224,218 surviving children aged 0–59 months, of whom 1.35% lived with their mother alone. The primary outcome variable was stunting, defined as a height-for-age Z-score below −2 standard deviations (SD) according to WHO growth standards. Descriptive statistics, logistic regression models (both unadjusted and sequentially adjusted for child-maternal-household, and community-level factors), and Fairlie decomposition analysis were employed to quantify the contribution of various factors to the observed disparities. The key finding revealed that children living with their mother alone had a higher prevalence of stunting (39.8%) compared to those living with both parents (35.43%). However, this association became statistically non-significant after adjusting for socioeconomic and demographic covariates in the fully adjusted model.

Previous studies have shown that single mothers often experience economic and social disadvantages, which increase the risk of undernutrition among their children (14, 28, 29) Our finding from the logistic regression models revealed a progressive attenuation of the association between living with mother alone and stunting. In the unadjusted model, children in mother-only households had 21% higher odds of being stunted. After adjusting for child-level characteristics such as age, sex, birth order, size at birth, and birth weight, the odds ratio decreased slightly to 17%, indicating that these early-life biological and demographic factors partially accounted for the disparity. Further adjustment for maternal characteristics—including education, BMI, height, antenatal care, and delivery place—reduced the odds ratio to 11%, suggesting that maternal health and human capital play a substantial mediating role. However, in the fully adjusted model, which included household wealth, caste, religion, region, and other contextual variables, the association was no longer statistically significant 7%. This indicates that the elevated risk of stunting among children in single-mother households is largely explained by their disadvantaged socioeconomic and structural circumstances rather than the absence of the father *per se*. These findings align with prior research showing that household poverty, maternal undernutrition, and low educational attainment are stronger predictors of child stunting than family structure alone Factors associated with child stunting, wasting, and underweight in 35 low-and middle-income countries (30, 31). For example, evidence from 137 developing countries showed that maternal undernutrition and infection, adolescent motherhood, short birth intervals, fetal growth restriction, preterm birth, and child undernutrition and infection—rather than single parenthood—were the main determinants of poor child growth outcomes (10). Similarly, evidence from Uganda Bangladesh suggests that maternal education and household wealth mediate much of the nutritional disadvantage faced by marginalized children (32, 33).

The Fairlie decomposition analysis further illuminated the mechanisms underlying the stunting gap between the two groups. When only child-level characteristics were considered, they explained 21.09% of the disparity, with child age being the dominant contributor This finding aligns with global evidence highlighting a critical window—from conception to the first 2 years of life (0–23 months)—within which nearly 70% of stunting occurs. Growth deficits tend to persist until the age of five, primarily due to ongoing exposure to modifiable risk factors related to nutrition, infections, and psychosocial care (34). However, once maternal characteristics were introduced, the explained portion of the gap nearly tripled to 55.12%, underscoring the pivotal role of maternal factors. Notably, maternal height contributed −25.56%, indicating that short maternal height is strongly associated with increased risk of child stunting because it reflects a mother’s own early-life undernutrition and infections, which limit her skeletal growth. Biologically, shorter mothers are more likely to experience intrauterine growth restriction, preterm delivery, and smaller birth size of infants. These disadvantages at birth persist into early childhood, raising the likelihood of stunting (35). Maternal education also played a major role (−22.27%), reinforcing the protective effect of women’s schooling on child nutrition, this finding aligns with existing evidence that educated mothers are better equipped with knowledge of appropriate feeding, healthcare-seeking, and hygiene practices, which translate into improved child growth outcomes. Moreover, schooling enhances women’s autonomy and decision-making capacity, allowing them to allocate household resources more effectively toward nutrition and health. Therefore, strengthening female education not only benefits women themselves but also has intergenerational advantages by reducing the risk of stunting among their children (36, 37). The negative contributions suggest that single-mother households are disadvantaged in these key maternal attributes, thereby driving much of the observed stunting gap.

In the final decomposition model, which included household and community-level variables, 70.19% of the stunting gap was explained, highlighting the overwhelming influence of structural and socioeconomic factors. The wealth index emerged as the single largest contributor (28.29%), underscoring the central role of household economic status in shaping child nutritional outcomes. Children from poorer households face multiple disadvantages, including inadequate access to diverse diets, poor living and sanitation conditions, and limited healthcare utilization, higher disease burden all of which heighten their risk of stunting. This finding highlights the need for poverty alleviation strategies and equitable access to health and nutrition services as critical interventions to reduce child undernutrition that collectively impair linear growth (33, 38). Social caste also contributed significantly (13.01%), reflecting persistent inequities rooted in India’s social hierarchy, children from Scheduled Castes and Tribes experience overlapping disadvantages of poverty, poor living conditions, limited education, and restricted access to health services, all of which heighten their risk of stunting (39, 40). Geographic variation, captured by region (−16.39%), further emphasized the role of place-based disparities, with Central and Eastern regions bearing a higher burden These states are marked by entrenched poverty, low maternal education, poor sanitation, high fertility, and weaker health infrastructure, which together sustain high levels of stunting (41). Interestingly, while maternal height remained a strong contributor (24.23%), its effect was now positive, suggesting that even after accounting for wealth and caste, the biological disadvantage associated with short maternal stature continues to exert a direct influence on child growth. The unexplained portion of the gap declined from 78.91% in the first model to 29.81% in the full model, indicating that observed socioeconomic and demographic factors account for the majority of the disparity. This is consistent with studies showing that structural inequalities—not individual or familial deficits—are the root causes of child undernutrition (42, 43).

### Policy implications

This study reveals that the higher stunting risk among children in mother-only households in India is not due to family structure itself, but to deep-seated structural inequities particularly poverty, maternal undernutrition, and caste-based disadvantage. This demands a shift in policy thinking: existing nutrition programs like POSHAN Abhiyaan must move beyond universal delivery and adopt equity-focused targeting (44). Currently, POSHAN Abhiyaan and ICDS provide supplementary nutrition, growth monitoring, and counseling, but they rarely identify or prioritize single-mother households a group often invisible in administrative data (45). To improve outcomes, Anganwadi workers should be trained to screen for household composition during registration and link single mothers to integrated support: conditional cash transfers (e.g., Pradhan Mantri Matru Vandana Yojana), maternal nutrition supplements, and mental health services (46).

Moreover, social protection must address root causes: expand widow pensions, ensure access to property rights, and strengthen childcare support to enable livelihood participation. Given that maternal height a marker of intergenerational deprivation—explains 24% of the gap, investments must begin earlier: adolescent nutrition programs (e.g., Kishori Shakti Yojana) should be intensified in high-stunting regions (47). Critically, policies must avoid stigmatizing single mothers as “deficient.” Instead, frame support as correcting systemic exclusion—not fixing families.

### Strengths and limitations

A major strength of this study is its use of nationally representative, high-quality data from NFHS-5, enabling robust estimation of stunting disparities among under-five children in India. The large sample size (*n* = 224,218) allowed for precise analysis of a relatively small but vulnerable group—children living with mothers alone (1.35%)—while comprehensive covariates enabled stepwise adjustment across multiple levels. The application of Fairlie decomposition provided unique insights into the drivers of inequality, revealing how structural factors like wealth, caste, and maternal height explain 70.19% of the stunting gap. However, the cross-sectional design limits causal inference, and unmeasured factors—such as paternal absence due to migration versus widowhood, household food insecurity, or maternal mental health—may influence results but were not captured. Social desirability bias may also lead to underreporting of single-mother households. Despite these limitations, findings offer critical evidence for addressing structural inequities in child nutrition. Future research should employ alternative methodologies, such as trend analysis using data from the last five rounds of the NFHS, to better capture the temporal dynamics of child undernutrition in the context of single motherhood. It should also examine gender-based nutritional disparities among children and distinguish between widowed, divorced, and separated mothers to assess how these different circumstances influence child undernutrition outcomes, including stunting, wasting, and underweight.

## Conclusion

The higher prevalence of stunting among under-five children living with their mother alone compared to those with both parents is not driven by single factor, but by the deep-rooted socioeconomic and structural disadvantages these households face. Regression analysis reveals that even after accounting for child, maternal and household characteristics stunting remains higher among children living with only their mother. The Fairlie decomposition confirms this: 70.19% of the stunting gap is explained by observable inequities, with household wealth (28.29%) and maternal height (24.23%) being the most powerful contributors. This depicts that poverty and maternal height are the true drivers of stunting in this vulnerable group. Children in mother-only households are more likely to belong to poorer, marginalized communities, have undernourished and less-educated mothers, and lack access to quality healthcare and sanitation. Therefore, policies targeting stunting must move beyond a narrow focus on family composition and instead address systemic inequities. Strengthening social protection programs, improving access to education and livelihoods for single mothers, and enhancing maternal and child nutrition services in disadvantaged regions are critical steps toward reducing stunting in India (48).

## Data Availability

This study is based on secondary analysis of anonymized, publicly available data from the National Family Health Survey (NFHS-5), 2019–21, conducted by the International Institute for Population Sciences (IIPS), Mumbai, India, with technical assistance from ICF, under the stewardship of the Ministry of Health and Family Welfare, Government of India. The survey protocol was approved by the IIPS Institutional Review Board (IRB) in accordance with international ethical standards, including the Declaration of Helsinki. The studies were conducted in accordance with the local legislation and institutional requirements. Written informed consent for participation was not required from the participants or the participants’ legal guardians/next of kin in accordance with the national legislation and institutional requirements.
